# What is the best first-line combination regimen for advanced EGFR-mutated non-small cell lung cancer: a network meta-analysis and systemic review

**DOI:** 10.3389/fphar.2025.1572115

**Published:** 2025-08-11

**Authors:** Chuang Yang, Yunfei Wang, Yiyong Sun, Ying Sun, Rongyu Zhang, Chengjun Wang, Yanan Song, Wen Zhao, Jisheng Li

**Affiliations:** ^1^ Department of Medical Oncology, Qilu Hospital, Cheeloo College of Medicine, Shandong University, Jinan, Shandong, China; ^2^ Department of Respiratory Medicine, People’s Hospital of Zhangqiu, Jinan, Shandong, China; ^3^ Department of Medical Oncology, Qilu Hospital (Qingdao), Cheeloo College of Medicine, Shandong University, Qingdao, Shandong, China; ^4^ Cheeloo College of Medicine, Shandong University, Jinan, Shandong, China

**Keywords:** non-small cell lung cancer, epidermal growth factor receptor tyrosine kinase inhibitors, combination regimens, efficacy, network meta-analysis

## Abstract

**Background:**

Despite significant survival improvements from third-generation epidermal growth factor receptor tyrosine kinase inhibitors (EGFR-TKIs) in patients with advanced EGFR-mutated non-small cell lung cancer (NSCLC), almost all patients eventually develop resistance. Currently, some studies have confirmed that combination therapy regimens based on third-generation EGFR-TKIs can further enhance efficacy. However, it remains unknown which specific combination regimen is more effective.

**Methods:**

Randomized clinical trials comparing combination treatments involving third-generation EGFR-TKIs vs. EGFR-TKI single agent for advanced EGFR-mutated NSCLC patients were included. The primary outcome was progression-free survival (PFS), while secondary outcomes included overall survival (OS), objective response rate (ORR) and treatment-related adverse events (TRAEs). Subgroup analyses were also conducted.

**Results:**

The study encompassed 5 trials, involving 1791 patients. The combination of osimertinib with chemotherapy and with ramucirumab, as well as the combination of lazertinib with amivantamab, have been shown to significantly improve PFS compared to osimertinib monotherapy. The combination of osimertinib with chemotherapy and the combination of lazertinib with amivantamab resulted in a significantly higher incidence of TRAEs compared to osimertinib monotherapy and other combination regimens. Osimertinib with chemotherapy showed better PFS in almost all the subgroup analyses. In patients with brain metastases, osimertinib plus chemotherapy appeared to offer clinical benefits over other treatment strategies, although statistical significance was only observed in comparison with osimertinib monotherapy.

**Conclusion:**

This network meta-analysis suggests osimertinib plus pemetrexed-based chemotherapy as the only regimen demonstrating PFS benefits in the whole cohort and almost all the subgroup analyses, making it the optimal treatment for patients with advanced EGFR-mutated NSCLC. Given the higher incidence of grade ≥3 TRAEs, careful consideration is needed in clinical practice.

**Systematic Review Registration:**

https://www.crd.york.ac.uk/prospero/, identifier, CRD42024579401.

## 1 Introduction

Lung cancer is the leading cause of cancer incidence and mortality worldwide, with non-small cell lung cancer (NSCLC) accounting for approximately 85% of cases (Siegel et al., 2024; Travis et al., 2015). Epidermal growth factor receptor (EGFR) mutations are observed in 15%–50% of non-squamous advanced NSCLC cases, particularly among women and non-smokers (Pao and Girard, 2011; Jänne and Johnson, 2006). EGFR tyrosine kinase inhibitors (EGFR-TKIs) significantly enhance survival in patients with advanced EGFR-mutated NSCLC, with osimertinib, a third-generation EGFR-TKI, demonstrating superior survival benefits over first-generation EGFR-TKIs (Soria et al., 2018). However, despite initial efficacy, most patients eventually develop resistance to osimertinib, after which platinum-based chemotherapy remains the standard treatment and the 3-year survival rate remains below 30% (Ramalingam et al., 2020). Additionally, 30%–50% of these patients develop central nervous system (CNS) metastases within 5 years of diagnosis, leading to poorer survival outcomes (Rangachari et al., 2015; Cho et al., 2022). Therefore, there is an urgent need for potent therapeutic options that can improve the objective response rate (ORR) and extend progression-free survival (PFS).

In the past decade, considerable efforts have been directed toward improving outcomes in EGFR mutation-positive NSCLC patients. When multiple therapeutic strategies demonstrate superiority over older standards of care, oncologists naturally seek to combine these newer therapies to further enhance patient outcomes (Meyer et al., 2024). Current combination strategies involve third-generation EGFR-TKIs paired with chemotherapy, anti-angiogenic agents, or mesenchymal-epithelial transition (MET) inhibitors, with MET amplification being the most common mechanism of acquired resistance to osimertinib (Meyer et al., 2024; Leonetti et al., 2019). While many of these combinations show improved efficacy compared to osimertinib alone, they are also associated with a higher incidence of treatment-related adverse events (TRAEs) and a broader spectrum of adverse effects (Soria et al., 2018; Cho et al., 2024; Motta-Guerrero et al., 2024). Moreover, interpreting survival data across different trials is challenging due to variations in study size, follow-up duration, and the availability of subsequent therapies at the time of disease progression. These factors limit the ability to compare the efficacy and toxicity of different approaches across trials. There is ongoing debate about whether all patients should receive combination treatments, and it remains crucial to identify which subgroups might benefit most from specific combination therapies, especially when considering TRAEs.

In this network meta-analysis (NMA) of randomized controlled trials, we aimed to provide a well-designed comparative synthesis of the relative efficacy and safety of all combination treatments involving third-generation EGFR-TKIs in patients with advanced EGFR-mutated NSCLC as first-line therapy. Additionally, we conducted a subgroup analysis to determine the optimal clinical choices for specific patient subgroups, with a particular focus on those with CNS metastases.

## 2 Methods

NMA enables the integration of both direct and indirect evidence, allowing for quantitative comparisons and ranking of multiple interventions. By constructing an interconnected network of available evidence, NMA addresses the lack of head-to-head trial data, thereby providing more comprehensive and robust support for clinical decision-making. This NMA was conducted following the Preferred Reporting Items for Systematic Reviews and Meta-Analyses (PRISMA) guidelines. Specifically, we adhered to the PRISMA Extension version for NMAs (PRISMA-NMA) as outlined in Supplementary Table S1. Additionally, the research project has been registered with the International Prospective Register of Systematic Reviews (PROSPERO) under the registration number CRD42024579401.

### 2.1 Retrieval method

We used the following search terms to identify relevant studies: osimertinib, aumolertinib, furmonertinib, befotertinib, lazertinib, non-small-cell lung carcinoma, ErbB receptors, and randomized controlled trial, along with their corresponding MeSH terms. The databases searched included PubMed, EMBASE, Web of Science and Cochrane Library. The detailed search strategy is provided in Supplementary Table S2.

### 2.2 Inclusion and exclusion criteria

Chuang Yang and Yunfei Wang independently conducted the searches and evaluations to determine the eligibility of each identified study. They assessed studies by screening titles and abstracts, with full texts reviewed when necessary. The inclusion criteria for screening studies were as follows: (1) clinical trials must be prospective, randomized, and controlled; (2) patients must have a confirmed diagnosis of NSCLC through histological or cytological analysis; (3) genetic testing must confirm the presence of EGFR mutations in the patients; (4) patients must not have received any prior antitumor treatments; and (5) the clinical trials must use third-generation EGFR-TKI targeted therapy as the initial treatment. The exclusion criteria were as follows: (1) clinical trials that were retrospective or single-arm studies; (2) studies that did not provide complete survival data; and (3) patients who were not tested for EGFR mutations.

### 2.3 Data extraction

The primary outcome measured was PFS. Secondary outcomes included overall survival (OS), ORR, and grade ≥3 TRAEs. The main data extracted included the hazard ratio (HR) with a 95% confidence interval (CI) for PFS and OS, as well as dichotomous data for ORR and grade ≥3 TRAEs. Additional data collected encompassed the country of origin, primary author, year of publication, drugs used in the studies, sample size, and subgroup information such as patient’s age and sex distribution, smoking status, history of brain metastases, and types of EGFR mutations. Data collection was conducted by two investigators, Chuang Yang and Yunfei Wang. Any discrepancies were resolved by consensus or, if needed, by the intervention of a third person, Jisheng Li.

### 2.4 Assessment of quality

The risk of bias and quality assessment of the included studies were independently evaluated by two authors using Review Manager 5.4 software. Data extraction was also carried out independently by two investigators, Chuang Yang and Yunfei Wang. Any disagreements were resolved through discussion or by consulting a third author.

### 2.5 Data analysis

The ‘R’ software (version 4.3.1) was used to compare the efficacy and safety of different treatments using the ‘gemtc’ R package in this network meta-analysis. Statistical significance was defined as a two-sided p-value <0.05. We set the burn-in sample size to 100,000 and the number of iterations to 150,000. A Bayesian fixed-effect consistency model was employed to compare various treatment combinations. Additionally, the likelihood of each intervention being ranked as the top choice for different outcomes was calculated using the ‘R’ software. The surface under the cumulative ranking (SUCRA) probabilities were plotted to present the comparisons of treatments for PFS, OS, ORR, and grade ≥3 TRAEs. Chuang Yang and Yunfei Wang made significant contributions to the statistical analysis.

## 3 Results

### 3.1 Basic information

**FIGURE 1:**
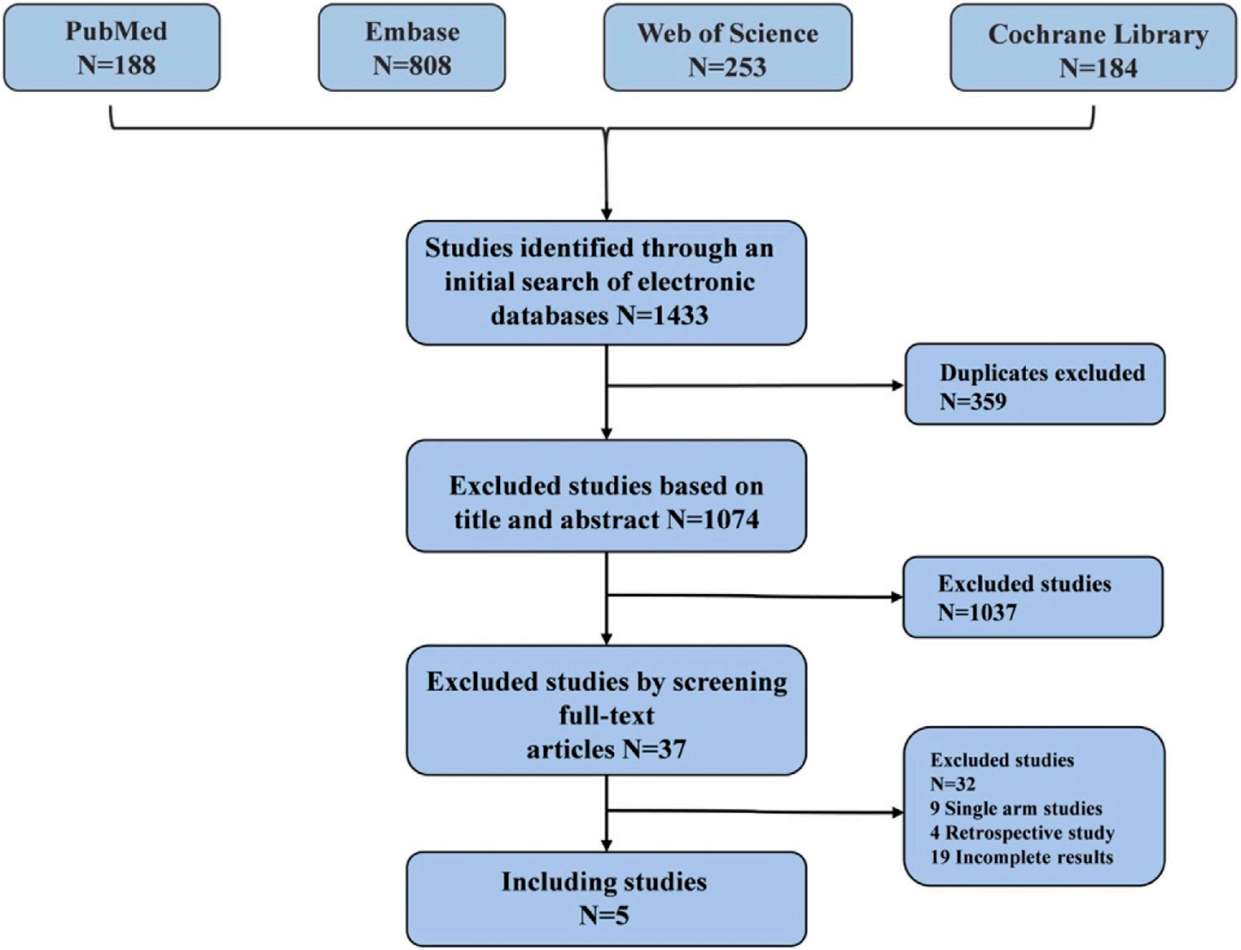
The flow chart of studies selection.

**FIGURE 2:**
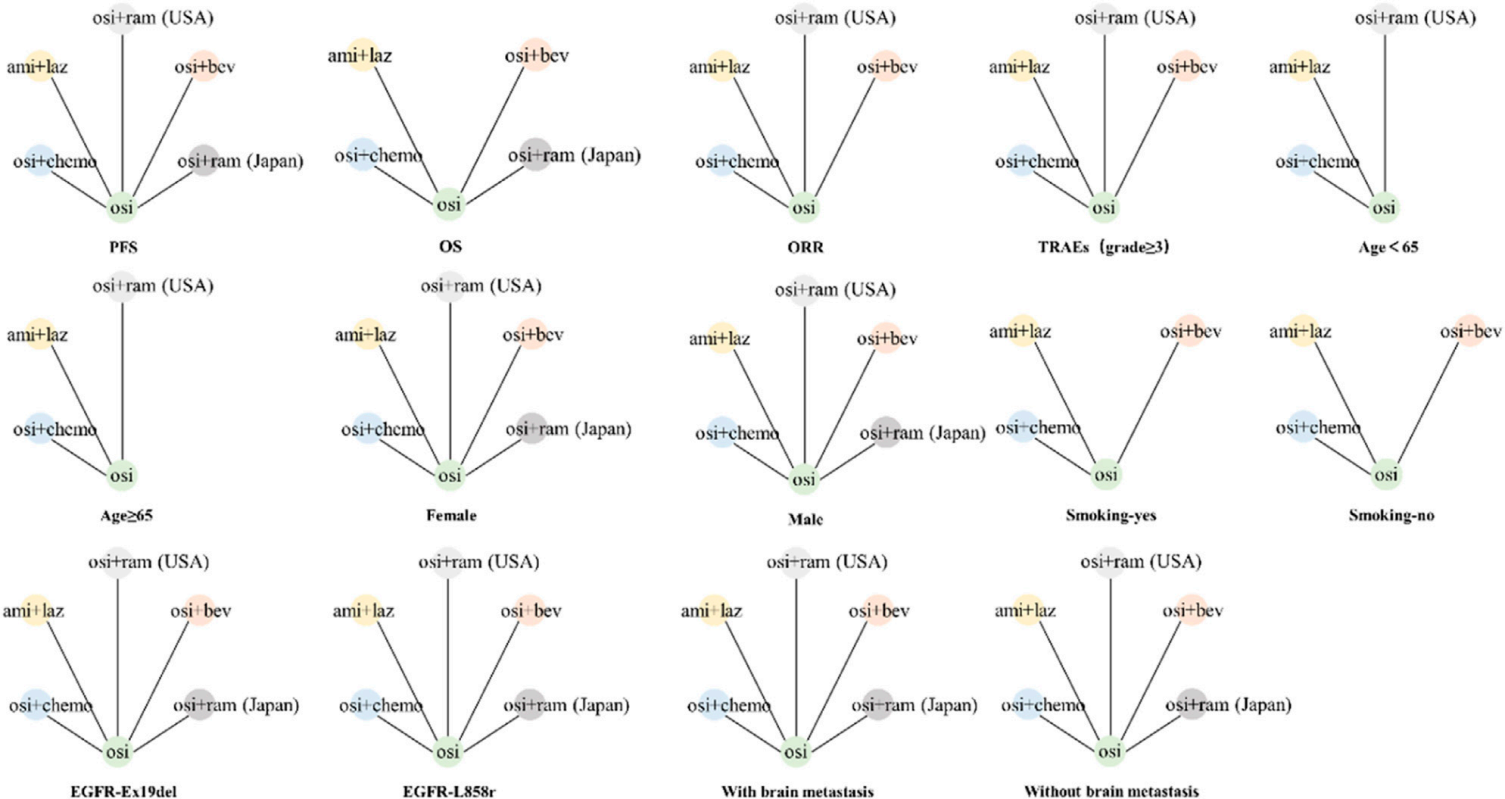
The network diagrams of comparisons in different groups including PFS, OS, ORR, grades≥3 TRAEs and different subgroups for patients with EGFR-mutated of NSCLC. The node represents treatment regimen, and the comparison between two treatments is connected by a line. PFS: progression-free survival; OS: overall survival; ORR: objective response rate; TRAEs: treatment-related adverse events; osi: osimertinib; osi + chemo: osimertinib + platinum-pemetrexed; ami + laz: amivantamab + lazertinib; osi + ram (United States): osimertinib + ramucirumab; osi + bev: osimertinib + bevacizumab; osi + ram (Japan): osimertinib + ramucirumab.

A comprehensive database search identified 1,433 studies: 188 from PubMed, 808 from Embase, 253 from Web of Science and 184 from Cochrane Library. Ultimately, 5 randomized controlled trials were deemed eligible for analysis (Figure 1). A total of 1,791 patients received one of five different treatments: osimertinib with chemotherapy, amivantamab with lazertinib, osimertinib with bevacizumab, osimertinib with ramucirumab, or osimertinib alone (Cho et al., 2024; Planchard et al., 2023; Kenmotsu et al., 2022; Le et al., 2023; Yoh et al., 2024). Two of these randomized controlled trials investigated osimertinib with ramucirumab: the RAMOSE trial from the United States (osimertinib + ramucirumab [United States]) and the OSIRAM-1 trial from Japan (osimertinib + ramucirumab [Japan]). We did not categorize these two studies under the same protocol due to differences in the application of ramucirumab between the two trials (Le et al., 2023; Yoh et al., 2024). Basic information about the included studies was presented in Table 1, while the network diagram was shown in Figure 2. The evaluation of bias, illustrated in Supplementary Figure S1, indicated a low risk of bias. The diagnostic and the density trace plot of PFS, OS, ORR and TRAEs, were shown in Supplementary Figure S3.

**TABLE 1:** The characteristics of studies enrolled in the network meta-analysis.

No.	Study	Country	Author	Year	Intervention arm	No. of Patients	Control arm	No. of Patients	PFS (HR; 95% CI)	OS (HR; 95% CI)
1	FLAURA2	-	D.Planchard.et al.	2023	Osimertinib plus platinum-pemetrexed	279	Osimertinib	278	0.62 (0.49–0.79)	0.75 (0.57.0.97)
2	MARIPOSA	Korea	B.C.Cho.et al.	2023	Amivantamab plus Lazertinib	429	Osimertinib	429	0.70 (0.58–0.85)	0.80 (0.61.1.05)
3	RAMOSE	United States	X.Le.et al.	2023	Osimertinib plus Ramucirumab	93	Osimertinib	46	0.55 (0.32–0.93)	-
4	WJOG9717L	Japan	H.Kenmotsu.et al.	2022	Osimertinib plus Bevacizumab	61	Osimertinib	61	0.862 (0.531–1.397)	0.970 (0.505.1.866)
5	OSIRAM-1	Japan	K.Yoh.et al.	2024	Osimertinib plus Ramucirumab	57	Osimertinib	58	1.054 (0.674.1.648)	1.159 (0.645.2.083)

No, number; OS, overall survival; PFS, progression-free survival; HR, hazard ratio; CI, confidence.

### 3.2 PFS analysis, OS analysis and ORR analysis

**FIGURE 3:**
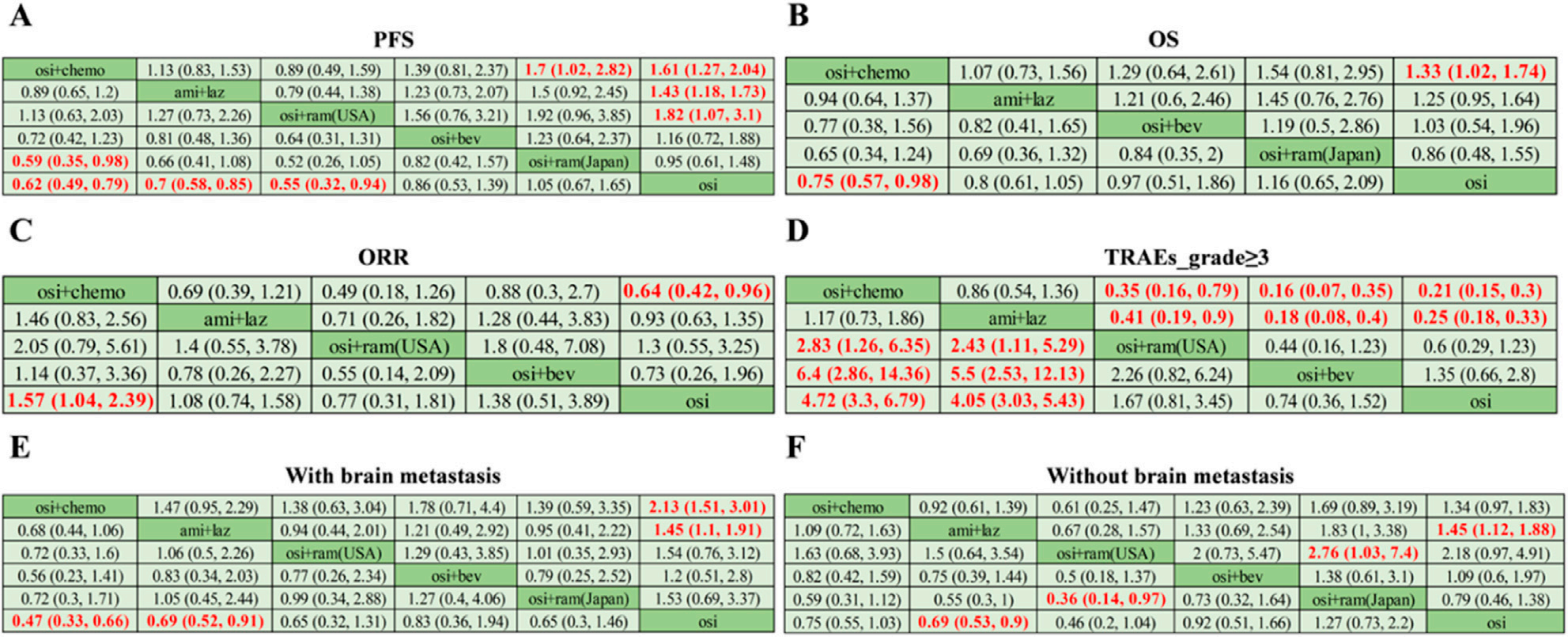
The pooled estimates of the network meta-analysis in patients with EGFR mutated of NSCLC. **(A)** HRs and 95% CI for progression-free survival (PFS); **(B)** HRs and 95% CI for overall survival (OS); **(C)** ORs and 95% CI for objective response rate (ORR); **(D)** ORs and 95% CI for grade≥3 treatment-related adverse events (TRAEs_grade≥3); **(E)** HRs and 95% CI for PFS in patients with brain metastasis; **(F)** HRs and 95% CI for PFS in patients without brain metastasis. The data marked in bold and red is statistically significant. osi: osimertinib; osi + chemo: osimertinib + platinum-pemetrexed; ami + laz: amivantamab + lazertinib; osi + ram (United States): osimertinib + ramucirumab; osi + bev: osimertinib + bevacizumab; osi + ram (Japan): osimertinib + ramucirumab.

**FIGURE 4:**
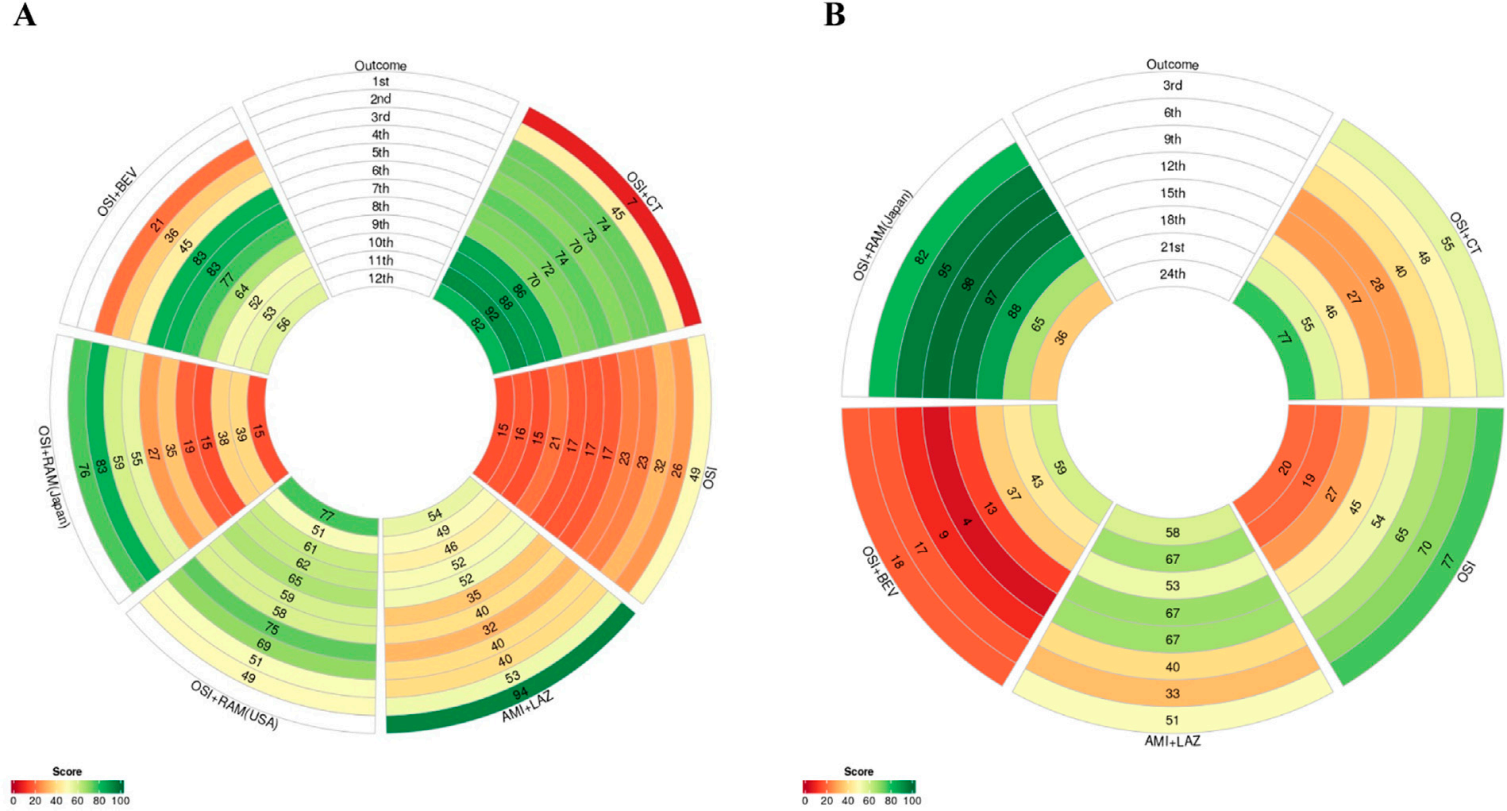
The rank-heat plot illustrating the evaluation of various regimens used as first-line treatment for patients with EGFR mutated of NSCLC. The overall ranking for each regimen and outcome were represented by the sectors in the figure that are color-coded according to the surface under the cumulative ranking (SUCRA) value. **(A)** SUCRA values for PFS; **(B)** SUCRA values for OS. The circles in the plot represent the SUCRA values for PFS at 1st, 2nd, 3rd, 4th, 5th, 6th, 7th, 8th, 9th, 10th, 11th, and 12th month, as well as SUCRA values for OS at 3rd, 6th, 9th, 12th, 15th, 18th, 21st, and 24th month for different treatments. osi: osimertinib; osi + chemo: osimertinib + platinum-pemetrexed; ami + laz: amivantamab + lazertinib; osi + ram (United States): osimertinib + ramucirumab; osi + bev: osimertinib + bevacizumab; osi + ram (Japan): osimertinib + .ramucirumab.

In terms of PFS (Figure 3A), osimertinib combined with chemotherapy demonstrated superior benefits compared to osimertinib alone (HR = 0.62; 95% CI: 0.49–0.79) and also showed significant advantages over osimertinib combined with ramucirumab (Japan) (HR = 0.59; 95% CI: 0.35–0.98). Additionally, the combination of amivantamab and lazertinib (HR = 0.70; 95% CI: 0.58–0.85) and osimertinib combined with ramucirumab (United States) (HR = 0.55; 95% CI: 0.32–0.94) significantly prolonged PFS compared to osimertinib alone. However, osimertinib with bevacizumab (HR = 0.86; 95% CI: 0.53–1.39) and osimertinib with ramucirumab (Japan) (HR = 1.05; 95% CI: 0.67–1.65) did not show a statistically significant benefit in PFS. By calculating the PFS rates of different combination treatments at the 1st through 12th months (Table 2), we found that osimertinib combined with chemotherapy (OR = 2.11; 95% CI: 1.44–3.11) and amivantamab combined with lazertinib (OR = 1.55; 95% CI: 1.16–2.07) significantly increased the 12-month PFS rate compared to osimertinib alone. Notably, osimertinib combined with chemotherapy showed superiority in PFS rates from the 3rd to the 12th month, except for the 5th month (OR = 1.81; 95% CI: 0.98–3.45), where the result was not statistically significant. Other regimens did not show a statistical significance in any time point. The PFS rate data were summarized in a matrix plot that compared the effectiveness of each treatment from the 1st to the 12th months (Supplementary Table S4). According to the Rank-Heat Plot, osimertinib combined with chemotherapy demonstrated the highest capacity for improving PFS, followed by osimertinib combined with ramucirumab (United States) and osimertinib with bevacizumab (Figure 4A).

**TABLE 2:** Comparing the PFS rate of different third-generation EGFR-TKI combination agents at the 1st, 2nd, 3rd, 4th, 5th, 6th, 7th, 8th, 9th, 10th, 11th and 12th month, with ORs and 95% CI.

Time (months)	osi + chemo	ami + laz	osi + ram (United States)	osi + bev	osi + ram (Japan)
1st	0.19 (0.01, 1.47)	2.36 (0.75, 8.99)	-	-	-
2nd	1.24 (0.5, 3.17)	1.36 (0.68, 2.75)	1.29 (0.15, 8.46)	-	3.77 (0.4, 105.75)
3rd	**2.05 (1.03, 4.23)**	1.09 (0.61, 1.97)	1.27 (0.15, 8.52)	0.4 (0.01, 5.27)	5.22 (0.64, 142.23)
4th	**1.99 (1.05, 3.91)**	1.19 (0.74, 1.91)	2.11 (0.46, 9.73)	0.99 (0.1, 9.49)	1.77 (0.39, 9.62)
5th	1.81 (0.98, 3.45)	1.08 (0.69, 1.71)	2.18 (0.63, 7.5)	1.28 (0.32, 5.68)	1.57 (0.41, 6.69)
6th	**2.03 (1.21, 3.45)**	1.26 (0.86, 1.85)	1.66 (0.55, 4.81)	2.93 (0.9, 11.49)	0.98 (0.28, 3.43)
7th	**1.91 (1.16, 3.2)**	1.18 (0.81, 1.72)	1.67 (0.55, 4.88)	2.94 (0.89, 11.7)	1.15 (0.38, 3.6)
8th	**1.74 (1.07, 2.87)**	1.4 (1, 1.97)	1.7 (0.66, 4.27)	2.09 (0.84, 5.4)	0.87 (0.32, 2.27)
9th	**2.07 (1.35, 3.23)**	1.33 (0.96, 1.87)	1.56 (0.62, 3.86)	1.63 (0.69, 4.04)	0.79 (0.31, 1.95)
10th	**2.13 (1.4, 3.31)**	1.29 (0.94, 1.77)	1.57 (0.66, 3.69)	1.41 (0.63, 3.23)	1.19 (0.5, 2.83)
11th	**2.25 (1.49, 3.46)**	1.31 (0.97, 1.77)	1.36 (0.59, 3.13)	1.41 (0.63, 3.23)	1.18 (0.5, 2.82)
12th	**2.11 (1.44, 3.11)**	**1.55 (1.16, 2.07)**	2.1 (0.97, 4.51)	1.6 (0.74, 3.54)	0.89 (0.39, 2.05)

Results with statistically significant difference are shown in bold. PFS: progression-free survival; ORs: odds ratios; CI: confidence intervals; osi: osimertinib; osi + chemo: osimertinib + platinum-pemetrexed; ami + laz: amivantamab + lazertinib; osi + ram (United States): osimertinib + ramucirumab; osi + bev: osimertinib + bevacizumab; osi + ram (Japan): osimertinib + ramucirumab.

Bold values indicate statistically significant values (p<0.05).

In terms of OS (Figure 3B), osimertinib combined with chemotherapy (HR = 0.75; 95% CI: 0.57–0.98) demonstrated superior trend compared to osimertinib alone. However, amivantamab combined with lazertinib (HR = 0.80; 95% CI: 0.61–1.05), osimertinib combined with bevacizumab (HR = 0.97; 95% CI: 0.51–1.86), and osimertinib combined with ramucirumab (Japan) (HR = 1.16; 95% CI: 0.65–2.09) did not prolong the OS when compared to the osimertinib alone. Analysis of the OS rates at the 3rd, 6th, 9th, 12th, 15th, 18th, 21st, and 24th months revealed that only osimertinib combined with chemotherapy (OR = 1.52; 95% CI: 1.03–2.26) significantly increased the 24-month OS rate (Table 3). Additionally, at both the 12th month (OR = 10.83; 95% CI: 1.59–299.34) and the 15th month (OR = 4.31; 95% CI: 1.19–21.56), osimertinib combined with ramucirumab (Japan) significantly improved the OS rate compared to osimertinib alone. Amivantamab combined lazertinib, and osimertinib with bevacizumab did not show a statistical significance in any time point. The OS rate data were summarized in a matrix plot comparing the effectiveness of each treatment from the 3rd to the 24th month (Supplementary Table S5). According to the Rank-Heat Plot for OS (Figure 4B), osimertinib combined with ramucirumab (Japan) was associated with the highest long-term overall survival benefit from the 6th to the 24th month, suggesting it is a potential best choice.

**TABLE 3:** Comparing the OS rate of different third-generation EGFR-TKI combination agents at the 3rd, 6th, 9th, 12th, 15th, 18th, 21st, and 24th month, with ORs and 95% CI.

Time (months)	osi + chemo	ami + Laz	osi + bev	osi + ram (Japan)
3rd	0.79 (0.29, 2.02)	0.76 (0.37, 1.56)	-	-
6th	0.76 (0.36, 1.56)	0.62 (0.34, 1.11)	-	-
9th	0.77 (0.4, 1.45)	0.78 (0.47, 1.27)	-	-
12th	0.68 (0.38, 1.2)	1.15 (0.75, 1.76)	0.11 (0, 0.82)	**10.83 (1.59, 299.34)**
15th	0.82 (0.48, 1.36)	1.23 (0.84, 1.8)	0.44 (0.09, 1.87)	**4.31 (1.19, 21.56)**
18th	1.12 (0.71, 1.78)	1.17 (0.84, 1.65)	0.99 (0.31, 3.15)	2.36 (0.77, 8.2)
21st	1.26 (0.83, 1.94)	1.37 (1, 1.88)	1.14 (0.42, 3.14)	1.5 (0.57, 3.99)
24th	**1.52 (1.03, 2.26)**	1.28 (0.96, 1.73)	1.36 (0.56, 3.4)	1.04 (0.42, 2.58)

Results with statistically significant difference are shown in bold. OS: overall survival; ORs: odds ratios; CI: confidence intervals; osi: osimertinib; osi + chemo: osimertinib + platinum-pemetrexed; ami + laz: amivantamab + lazertinib; osi + bev: osimertinib + bevacizumab; osi + ram (Japan): osimertinib + ramucirumab.

Bold values indicate statistically significant values (p<0.05).

In terms of ORR (Figure 3C), the addition of chemotherapy to osimertinib (OR = 1.57; 95% CI: 1.04–2.39) significantly improved ORR compared to osimertinib alone. While osimertinib combined with bevacizumab (OR = 1.38; 95% CI: 0.51–3.89) also showed a better ORR compared to osimertinib alone, the result was not statistically significant. No significant difference was observed with other third-generation TKI-based combination treatments. Consequently, osimertinib combined with chemotherapy appears to be the most effective treatment for achieving a higher ORR.

### 3.3 Safety analysis

To assess the safety of various treatments, we analyzed the incidence of grade 3 or higher TRAEs (Figure 3D). The results indicated that osimertinib combined with chemotherapy (OR = 4.72; 95% CI: 3.30–6.79) and amivantamab combined with lazertinib (OR = 4.05; 95% CI: 3.03–5.43) were associated with a higher incidence of grade 3 or higher TRAEs compared to osimertinib alone. Conversely, osimertinib combined with bevacizumab (OR = 0.74; 95% CI: 0.36–1.52) was associated with a lower incidence of grade 3 or higher TRAEs compared to osimertinib alone, though the difference was not statistically significant. Additionally, we summarized the discontinuation rates due to adverse events associated with different treatment regimens (Supplementary Table S6). Among the intervention arms, osimertinib combined with ramucirumab (Japan) exhibited the highest discontinuation rate at 76%, whereas the combination of osimertinib and ramucirumab (United States) showed the lowest rate at 9.7%. In the control arms, the OSIRAM-1 study reported the highest discontinuation rate at 19%.

### 3.4 Subgroup analysis

This network meta-analysis was performed to examine the impact of age, gender, smoking status, EGFR mutation type, and brain metastasis on PFS. Supplementary Figure S2 presented a ranking profile for the efficacy and safety of the six treatment options across the overall population and various subgroups.

In patients with brain metastases (Figure 3E), osimertinib combined with chemotherapy (HR = 0.47; 95% CI: 0.33–0.66) and amivantamab combined with lazertinib (HR = 0.69; 95% CI: 0.52–0.91) were superior to osimertinib alone with statistically significant. In patients without brain metastases (Figure 3F), amivantamab combined with lazertinib (HR = 0.69; 95% CI: 0.53–0.90) showed a statistically significant improvement in PFS compared to osimertinib alone. And osimertinib combined with ramucirumab (Japan) (HR = 0.46; 95% CI: 0.20–1.04) exhibited the lowest hazard ratio, though the difference was not statistically significant. Additionally, osimertinib combined with ramucirumab (United States) (HR = 0.36; 95% CI: 0.14–0.97) demonstrated a statistically significance compared to osimertinib combined with ramucirumab (Japan).

In patients aged <65 years (Supplementary Table S3A), both osimertinib combined with chemotherapy (HR = 0.59; 95% CI: 0.44–0.80) and amivantamab combined with lazertinib (HR = 0.50; 95% CI: 0.39–0.65) significantly improved PFS compared to osimertinib alone. In contrast, among patients aged ≥65 years (Supplementary Table S3B), only osimertinib combined with chemotherapy (HR = 0.68; 95% CI: 0.47–0.98) showed a statistically significant prolongation of PFS compared to osimertinib alone. Osimertinib combined with ramucirumab (United States) demonstrated a trend toward better PFS compared to osimertinib alone in both age groups (<65 years: HR = 0.56; 95% CI: 0.25–1.26; ≥65 years: HR = 0.55; 95% CI: 0.26–1.17), though these differences were not statistically significant.

In female patients (Supplementary Table S3C), osimertinib combined with chemotherapy (HR = 0.67; 95% CI: 0.49–0.92), amivantamab combined with lazertinib (HR = 0.70; 95% CI: 0.55–0.89), and osimertinib combined with ramucirumab (United States) (HR = 0.46; 95% CI: 0.24–0.87) all demonstrated significantly improved PFS compared to osimertinib alone. In male patients (Supplementary Table S3D), osimertinib combined with chemotherapy (HR = 0.54; 95% CI: 0.37–0.78) and amivantamab combined with lazertinib (HR = 0.74; 95% CI: 0.55–0.99) also showed a better PFS than osimertinib alone. Furthermore, osimertinib combined with chemotherapy (HR = 0.36; 95% CI: 0.17–0.78) was superior to osimertinib combined with ramucirumab (Japan).

For patients with a smoking history (Supplementary Table S3E), the combination of osimertinib and chemotherapy (HR = 0.63; 95% CI: 0.42–0.94) provided better PFS compared to osimertinib alone. Additionally, osimertinib combined with bevacizumab (HR = 0.48; 95% CI: 0.23–1.01) showed a trend toward better PFS compared to osimertinib in patients with a smoking history. In patients without a smoking history (Supplementary Table S3F), both osimertinib combined with chemotherapy (HR = 0.61; 95% CI: 0.46–0.82) and amivantamab combined with lazertinib (HR = 0.67; 95% CI: 0.53–0.85) demonstrated significantly improved PFS compared to osimertinib alone.

Regarding the most common types of EGFR mutations, we conducted a subgroup analysis. Among patients with EGFR 19del (Supplementary Table S3G), osimertinib combined with ramucirumab (United States) provided the best PFS benefit (HR = 0.49; 95% CI: 0.26–0.92), followed by osimertinib combined with chemotherapy (HR = 0.60; 95% CI: 0.44–0.82) and amivantamab combined with lazertinib (HR = 0.65; 95% CI: 0.50–0.84). Osimertinib combined with ramucirumab (United States) (HR = 0.39; 95% CI: 0.17–0.92), osimertinib combined with chemotherapy (HR = 0.48; 95% CI: 0.25–0.93), and amivantamab combined with lazertinib (HR = 0.53; 95% CI: 0.28–0.97) also demonstrated statistically significant superiority compared to osimertinib combined with ramucirumab (Japan). Among patients with EGFR L858R (Supplementary Table S3H), only osimertinib combined with chemotherapy (HR = 0.63; 95% CI: 0.44–0.90) showed improved efficacy compared to osimertinib alone. However, osimertinib combined with ramucirumab (United States) showed a better effect compared to osimertinib alone but the result was not statistically significant.

## 4 Discussion

Although most patients with advanced EGFR-mutated NSCLC initially respond to treatment with third-generation EGFR-TKIs, real-world survival estimates indicated that only 19% of patients were alive after 5 years (Bazhenova et al., 2021). This underscores the ongoing need to enhance clinical outcomes with first-line treatments beyond what has been achieved with EGFR-TKI monotherapy, especially since 25% of patients die before receiving second-line therapy (Nieva et al., 2022). In this systematic review and network meta-analysis, we comprehensively summarized the comparative efficacy and safety of multiple first-line combination strategies based on third generation EGFR-TKIs in patients with advanced EGFR mutated NSCLC. These results suggested that:

• The combination of osimertinib with chemotherapy and with ramucirumab, as well as the combination of lazertinib with amivantamab, have been shown to significantly improve PFS compared to osimertinib monotherapy.
• The combination of osimertinib with chemotherapy resulted in a significantly higher incidence of TRAEs compared to osimertinib combined with bevacizumab, ramucirumab, and osimertinib monotherapy. Similarly, the combination of lazertinib with amivantamab also showed a higher incidence of TRAEs; however, there is no statistically significant difference between these two combination regimens.
• Osimertinib with chemotherapy showed better PFS than osimertinib single agent in almost all the subgroup analysis except for patients without brain metastasis. Given the significantly increased incidence of TRAEs of osimertinib combined with chemotherapy, careful consideration should be given when selecting treatment regimens for different patient populations.

In this research, we selected PFS as the primary endpoint to more accurately evaluate the benefit of first-line therapy, as OS is influenced by the effects of all subsequent lines of treatment. Our analysis found that the combination of osimertinib with chemotherapy, osimertinib with ramucirumab, and lazertinib with amivantamab all extended PFS compared to osimertinib monotherapy. Below, we outlined the rationale for these three combination strategies and explored their potential mechanisms.

Although third-generation EGFR-TKIs initially demonstrate good efficacy and tolerance, nearly all patients eventually develop resistance. Platinum-based chemotherapy remains the standard of care for patients with EGFR-mutated advanced NSCLC who progress after third-generation EGFR-TKI treatment. Additionally, some patients may not respond to EGFR-TKIs from the outset. Research has indicated that concomitant gene mutations, such as TP53, are associated with higher rates of primary resistance to third-generation EGFR-TKIs (Seto et al., 2014; Yamamoto et al., 2021; Saito et al., 2019), and chemotherapy may help address these resistant clones. Therefore, combining third-generation EGFR-TKIs with chemotherapy could potentially overcome intratumoral heterogeneity by targeting both primary resistant and acquired resistant cell populations, thereby improving clinical outcomes. Our analysis supported this, showing that the combination of osimertinib with chemotherapy provided superior PFS compared to osimertinib alone across the overall population and in nearly all subgroups.

MET amplification and EGFR pathway re-activation are the most common acquired resistance to the third-generation EGFR-TKIs (Schoenfeld et al., 2020; Oxnard et al., 2018). Amivantamab, an EGFR-MET bispecific antibody, not only inhibits EGFR and MET signaling but also has immune cell-directing activity (Moores et al., 2016; Vijayaraghavan et al., 2020; Yun et al., 2020). This establishes a rationale for combining amivantamab with lazertinib, a highly selective, CNS-penetrant third-generation EGFR-TKI known for its efficacy against EGFR mutations and T790M resistance (Yun et al., 2019; Ahn et al., 2019). The combination of amivantamab and lazertinib may extend PFS compared to osimertinib alone by targeting MET amplification clones and modulating the tumor microenvironment, potentially leading to greater tumor shrinkage.

Extensive preclinical data highlight the relevance of anti-angiogenic strategies in patients with EGFR mutations. Bevacizumab, an anti-vascular endothelial growth factor (VEGF) monoclonal antibody, has been shown to reduce tumor vascular permeability, thereby improving drug delivery and increasing drug concentration within tumors (Wildiers et al., 2003; Dickson et al., 2007; Le et al., 2021). Previous studies demonstrated that combining the first-generation EGFR-TKI erlotinib with bevacizumab resulted in superior PFS compared to erlotinib alone in advanced NSCLC patients with EGFR mutations (Seto et al., 2014; Yamamoto et al., 2021; Saito et al., 2019; Kawashima et al., 2022; Stinchcombe et al., 2019). However, in our analysis, osimertinib plus bevacizumab did not show a significant improvement in PFS over osimertinib alone. The negative results observed in the WJOG9717L study might be attributed to the shorter duration of bevacizumab treatment (median 33.4 weeks) (Saito et al., 2019), compared to other trials where a median of 18 cycles of bevacizumab was administered (Yamamoto et al., 2021). Ramucirumab, a fully human monoclonal antibody targeting VEGFR-2, blocks downstream signaling that promotes angiogenesis and reduces blood supply to tumors. We included two trials investigating the efficacy of osimertinib plus ramucirumab as first-line treatment for EGFR-mutated advanced NSCLC. Due to variations in ramucirumab administration, we analyzed them separately. Osimertinib plus ramucirumab (United States) demonstrated a PFS benefit compared to osimertinib alone, whereas osimertinib plus ramucirumab (Japan) did not. This difference may be attributed to several factors. First, regarding the dosing schedules: in the RAMOSE study, patients received osimertinib (80 mg daily) combined with ramucirumab (10 mg/kg) every 3 weeks, whereas in the OSIRAM-1 study, ramucirumab was administered every 2 weeks. The more frequent administration of ramucirumab in the OSIRAM-1 study may have contributed to a higher incidence of TRAEs, ultimately impacting treatment efficacy. In this cohort, ramucirumab was discontinued in 45 patients (76%) due to adverse events, and osimertinib was discontinued in 12 patients (19%), compared to only 4 patients (8.6%) in the U.S. cohort. Second, the OSIRAM-1 study was conducted during the COVID-19 pandemic, which may have negatively affected treatment adherence and continuity. Additionally, there were significant differences in baseline characteristics between the two studies: in the OSIRAM-1 treatment group, 79.7% of patients had brain metastases, compared to 43.0% in the RAMOSE group. These differences in treatment regimens, adverse event profiles, and patient characteristics may collectively explain the divergent outcomes observed between the two trials. (Le et al., 2023; Yoh et al., 2024). Overall, our findings suggested that combining osimertinib with a VEGF or VEGFR inhibitor could be an effective strategy for EGFR-mutated advanced NSCLC patients, though optimal administration of anti-angiogenic agents warrants further investigation.

Osimertinib combined with chemotherapy showed OS benefit trend. Previous research indicated that concurrent mutations in other oncogenic drivers, such as TP53, PIK3CA, BRAF, MET, MYC, CDK6, and CTNNB1, were associated with higher rates of primary resistance to EGFR-TKIs and reduced efficacy of these treatments (Blakely et al., 2017; De Pas et al., 2011; Massarelli et al., 2013) and also reduced response to EGFR-TKIs (Barnet et al., 2017; Hong et al., 2018). Therefore, the addition of chemotherapy may overcome intratumor heterogeneity by targeting diverse cell populations and enhancing overall clinical outcomes. This rationale may help explain why osimertinib combined with chemotherapy showed a survival benefit. However, according to the design of the FLAURA2 trial, the alpha value for the second OS analysis was set at 0.000001. As a result, although there was a trend toward improved OS with the combination therapy, it did not reach statistical significance. Further follow-up is required to determine whether the addition of chemotherapy to osimertinib confers a meaningful OS benefit. Additionally, patients often receive multiple lines of treatment upon disease progression, making it essential to consider the efficacy of subsequent salvage therapies. This consideration underscores the importance of using PFS as the primary endpoint in our analysis to accurately assess the benefits of first-line treatments.

To optimize management and treatment selection, it is essential to understand the predominant adverse events associated with each EGFR-TKI combination during long-term use, as toxicity is a critical component of therapeutic evaluation. Our analysis revealed that osimertinib combined with chemotherapy resulted in a significantly higher incidence of grade 3 or higher TRAEs compared to osimertinib combined with bevacizumab, ramucirumab, or osimertinib alone. Similarly, the combination of lazertinib with amivantamab was also associated with a higher incidence of grade 3 or higher TRAEs; however, there was no statistically significant difference in TRAE incidence between these two combination therapies. For patients in poor physical condition, these combinations may not be appropriate due to the increased incidence of severe TRAEs. Furthermore, the spectrum of TRAEs varies between these combinations. In the FLAURA2 trial, 64% of patients receiving osimertinib combined with chemotherapy experienced grade 3 or higher TRAEs, with hematologic toxic effects affecting 71% of this group, interstitial lung disease or pneumonitis occurring in 3%, and cardiac effects reported in 9% (Planchard et al., 2023). In contrast, the combination of lazertinib with amivantamab, as reported in the MARIPOSA trial, resulted in 75% of patients experiencing grade 3 or higher TRAEs, with hypoalbuminemia (15%) and paronychia (11%) being the most common (Cho et al., 2024). The significant toxicity associated with these combinations raises the question of whether the gains in PFS justify the associated risks. While these regimens may not be suitable for all patients, particularly those with a high burden of disease or significant comorbidities, they may benefit specific high-risk subgroups. Therefore, treatment regimens should be selected based on the patient’s overall physical condition and individual comorbidities.

Previous studies have demonstrated that the combination of therapies can offer significant benefits over monotherapy, particularly in patient subgroups with high unmet needs, such as those with CNS metastases—an indicator of poorer prognosis (Bhatt et al., 2017; Peters et al., 2016; Lee et al., 2017). Given this, we investigated the efficacy of various combination regimens specifically in subgroups with CNS metastases. CNS metastasis is common among patients with EGFR-mutated advanced NSCLC and is associated with diminished prognosis and quality of life (Passaro et al., 2019). Approximately 30%–50% of these patients develop CNS metastases within 5 years of diagnosis (Rangachari et al., 2015; Cho et al., 2022), and this incidence is increasing due to improved survival rates and enhanced detection methods (Popat et al., 2023; Schoenmaekers et al., 2020). Third-generation EGFR-TKIs, such as osimertinib and lazertinib, exhibit potent blood-brain barrier penetration and significantly reduce the risk of CNS progression or death (Reungwetwattana et al., 2018; Goss et al., 2018; Ballard et al., 2016; Colclough et al., 2021; White et al., 2021). Despite this efficacy, outcomes for patients with pre-existing CNS metastases remain less favorable compared to those without (Soria et al., 2018). While local therapies such as neurosurgery or radiotherapy can be used for CNS metastases, they may also result in long-term cognitive deficits (Monaco et al., 2013; Parsons et al., 2021). Therefore, strategies that enhance CNS efficacy could potentially delay the need for local therapies in patients with asymptomatic brain metastases, thus preserving neurocognitive function and improving quality of life. In our analysis, both osimertinib combined with chemotherapy and lazertinib combined with amivantamab showed improved PFS compared to osimertinib alone in NSCLC patients with brain metastases. Previous research supports the use of EGFR-TKI and chemotherapy combinations in these patients, with studies showing better outcomes with carboplatin-pemetrexed plus gefitinib compared to gefitinib alone in EGFR-mutated advanced NSCLC, including those with baseline CNS metastases (Hosomi et al., 2020; Noronha et al., 2020; Hou et al., 2023). While pharmacokinetic studies suggest limited CNS penetration for cisplatin, carboplatin, and pemetrexed (Jacobs et al., 2005; Barlesi et al., 2011), chemotherapy has still demonstrated efficacy in the brain. For instance, in the AURA3 study, a CNS ORR of 17% was observed with the platinum-pemetrexed regimen in patients with baseline CNS metastases (Wu et al., 2018). Furthermore, a single-arm phase II trial reported a CNS ORR of 42% with cisplatin plus pemetrexed in 43 patients with NSCLC and brain metastases (Barlesi et al., 2011). It is possible that CNS metastases might facilitate the brain penetration of chemotherapy by disrupting the blood-brain barrier, thereby enhancing the synergistic effect of platinum-pemetrexed on the CNS efficacy of osimertinib. Similarly, lazertinib has shown promise in inhibiting CNS metastases, given the frequent occurrence of brain metastases in patients with EGFR-mutated NSCLC. A comparable improvement was observed among patients with a history of brain metastases who had not previously received brain radiation, reinforcing this conclusion (Passaro et al., 2024). The mechanism by which amivantamab improves intracranial PFS may involve either direct antitumor effects or immune-based mechanisms. However, further studies are needed to assess long-term neurocognitive outcomes to fully understand the impact of contemporary treatment strategies on brain function.

Existing evidence suggests that different types of EGFR mutations vary significantly in their clinical and pathological correlations, downstream signaling pathways, and responsiveness to EGFR-TKIs. Specifically, the benefit of EGFR-TKIs, particularly third-generation EGFR-TKIs, appears to be greater in tumors with exon 19 deletions compared to those with L858R mutations (Lee et al., 2015; Riely et al., 2006). Consequently, patients with L858R mutations may require combination treatments, as third-generation EGFR-TKIs alone may not provide sufficient efficacy for these individuals. In our analysis, we found that only osimertinib combined with chemotherapy demonstrated a significant improvement in PFS compared to osimertinib monotherapy in patients with L858R mutations. Additionally, we examined the efficacy of different treatment regimens based on factors such as age, gender, and smoking history. A ranking model was developed to identify the most effective combination strategy for specific subgroups.

The present study has several limitations. Firstly, as an observational network meta-analysis based solely on clinical trial data, it is subject to unavoidable confounding factors. Most treatments were compared indirectly, with direct evidence predominantly sourced from a single trial within the network. Consequently, the estimates should be interpreted cautiously due to their reliance on previous distributions and assumptions of transitivity and consistency, despite the inclusion of only randomized controlled trials and thorough investigation of these assumptions. Secondly, the use of OS as an endpoint might introduce heterogeneity, as it may not accurately reflect the effect of each treatment due to subsequent lines of therapy received after progression from front-line treatment. This limitation can affect the interpretation of OS data. Therefore, PFS was reported as the primary outcome measure, as it represents the period until first disease progression, independent of later-line treatments. Thirdly, OS data was immature and should be interpreted with caution. Additionally, due to the COVID-19 pandemic, patients in the osimertinib plus ramucirumab (Japan) study experienced shorter exposure to ramucirumab because they could not attend hospitals according to protocol. Finally, due to the relatively small sample sizes of the WJOG9717L and OSIRAM-1 studies, there may be an increased risk of bias. As such, the comparative efficacy of osimertinib in combination with bevacizumab or ramucirumab *versus* other treatment strategies should be interpreted with caution.

This review synthesized evidence from randomized controlled trials to provide clinicians with a reference for evaluating the strengths and weaknesses of various treatment options. Complementing recent guidelines, these findings addressed which combination treatments offered the best care for advanced NSCLC patients with activating EGFR mutations. Our network meta-analysis identified osimertinib plus pemetrexed-based chemotherapy as the only regimen demonstrating PFS benefits across all subgroups except for patients without brain metastases. Given the higher incidence of grade 3 or higher TRAEs, careful consideration is needed when selecting combinations for specific subgroups. Future trials comparing osimertinib plus pemetrexed-based chemotherapy directly with lazertinib plus amivantamab are warranted, particularly in light of emerging drug resistance challenges.

## Data Availability

All files and manuscript data are available, by contacting lijisheng@sdu.edu.cn.
